# Respiratory symptoms in Ecuadorian workers associated with informal labor and cantonal satellite pollution metrics

**DOI:** 10.3389/fpubh.2026.1874197

**Published:** 2026-07-22

**Authors:** Juan Pablo Piedra, Marco Erazo-Vallejo, Ketty Pinargote-Cedeño, Rossana Mendoza-Lopez, Bernarda Espinoza-Castro, Maria Teresa Solís-Soto

**Affiliations:** 1 Facultad de Ciencias Médicas, Universidad de las Américas, Quito, Ecuador; 2 Center for Translational Research in Oncology, Instituto do Câncer do Estado de São Paulo, São Paulo, Brazil; 3 CIH-LMU Center for International Health, LMU University Hospital Munich, Munich, Germany; 4 Institute and Clinic for Occupational, Social, and Environmental Medicine, LMU University Hospital Munich, Munich, Germany; 5 OH TARGET Competence Center, Universidad San Francisco Xavier, Sucre, Bolivia

**Keywords:** informal sector, occupational health, respiratory symptoms, satellite telemetry, sentinel-5p

## Abstract

**Introduction:**

Evidence on the association between ambient pollution and occupational hazards in Ecuador remains limited. This cross-sectional study evaluated the association between working conditions, satellite-derived ambient pollutants, geographic factors, and respiratory symptoms.

**Methods:**

We analyzed 1,807 workers across four sectors. Respiratory symptoms (ECRHS) were cross-referenced with Sentinel-5P telemetry, utilizing z-score standardized environmental variables and selecting optimal spatial/temporal concentration percentiles (p25, p50, p75, and p95) via the Akaike Information Criterion (AIC) for each outcome. Multivariable penalized logistic regression (Firth’s method) identified factors associated with respiratory symptoms.

**Results:**

Informal employment showed the strongest association with respiratory symptoms, including asthma (aOR 12.56; 95% CI: 5.09–32.87), wheezing (aOR 6.78; 95% CI: 1.83–27.89), chronic cough (aOR 4.14; 95% CI: 2.26–7.74), and chronic phlegm (aOR 2.53; 95% CI: 1.39–4.67). Direct workplace chemical inhalation, compounded by a complete lack of risk awareness, was associated with significantly higher odds for asthma, wheezing, and chronic cough. Geospatially, after controlling for canton-level altitude—which acted as an independent protective factor against asthma (aOR 0.78; 95% CI: 0.66–0.93)—nitrogen dioxide (NO2) and sulfur dioxide (SO2) emerged as the primary independent environmental predictors for chronic phlegm and cough, while ozone lost statistical significance.

**Discussion/conclusion:**

Informal employment and unprotected chemical inhalation are strongly associated with respiratory symptoms, frequently exceeding the risks observed in traditional high-exposure sectors. Furthermore, regional atmospheric pollution showed a significant statistical association with chronic respiratory outcomes at the cantonal level. Integrating high-resolution aerospace monitoring into occupational health surveillance represents a potential strategy to address these overlapping structural vulnerabilities; however, causal inference remains limited by the cross-sectional design.

## 1 Introduction

Respiratory diseases represent one of the leading causes of morbidity and mortality worldwide, with an estimated 262 million people suffering from asthma (1) and approximately 3.2 million deaths annually from chronic obstructive pulmonary disease (COPD), accounting for 74.4 million disability-adjusted life years (DALYs) (2, 3). In the Region of the Americas, these conditions, including those of occupational origin, represent a significant cause of death and disability, exacerbated by factors such as smoking, environmental pollution, and occupational exposure (4, 5). These diseases, which are strongly associated with environmental and occupational factors, constitute a growing public health problem. In this context, air pollution is one of the main challenges for public and occupational health worldwide, given its role in increasing the global burden of disease (6). The primary air pollutants include suspended particles (PM_2.5_ and PM_10_), nitrogen oxides such as nitrogen dioxide (NO_2_), ground-level ozone (O_3_), carbon monoxide (CO), and various volatile organic compounds (VOCs), generated mainly by human activities such as fossil fuel combustion in transport and industry, biomass burning, deforestation processes, and agricultural activities (7). In 2019, exposure to PM_2.5_ caused 4.1 million deaths worldwide, which were associated mainly with respiratory diseases (8).

Air pollution has been linked to various health effects among workers, especially in the informal sector. For example, high exposure to PM_2.5_ and PM_10_ has been reported during burning and dismantling activities at electronic waste recycling sites in Ghana (9), and in activities such as drilling, excavation, and roofing among construction workers in Iran (10). Exposure to environmental pollutants has also been linked to asthma in farmers and obstructive ventilatory dysfunction (OVD) in workers in China (11). In this study, a significant association was found between sulfates, nitrates, ammonium carbonate, organic matter, and black carbon and OVD, especially in women and young workers.

Considering the limitations of capturing workers’ environmental exposures both in the workplace and at their homes, several tools have been developed to capture different environmental exposures at various scales over time. In this sense, the TROPOMI (Tropospheric Monitoring Instrument) (12), onboard the European Space Agency’s Sentinel-5P satellite, stands out, as it allows the monitoring of atmospheric pollutants such as NO_2_, SO_2_, O_3_, CO, HCHO, CH_4_, and aerosols with high temporal and spatial resolution (13). One of the indicators includes the absorbing aerosol index (AAI), which detects atmospheric aerosols such as mineral dust, smoke from forest fires, and biomass burning (14, 15). For instance, Sentinel-5P data have been successfully used to evaluate variations in NO_2_ during COVID-19 lockdowns in Europe, validating satellite- derived retrievals against urban monitoring networks (16). Likewise, in Latin America, this tool has been used to monitor PM_2.5_ concentrations in Paraguay (17) and Ecuador, demonstrating its usefulness especially in contexts with limited monitoring infrastructure (18).

Although the association between occupational exposures and respiratory symptoms has been widely documented, few studies have integrated the evaluation of environmental pollutants using satellite data in working populations. This knowledge gap limits the understanding of how socio-occupational vulnerabilities and macro-environmental exposures concurrently relate to respiratory health outcomes. In this context, the objective of this study was to evaluate the association between exposure to air pollutants and the presence of respiratory symptoms in workers from different productive sectors in Ecuador. Addressing this gap through an integrated framework, consistent with the One Health approach, is essential to capture the complex intersection between human labor ecosystems and broader atmospheric environments.

## 2 Methods

### 2.1 Study design, population, and sample

We conducted a cross-sectional study based on a secondary analysis of the 2021-2022 National Survey of Workers’ Health in Ecuador, executed by the Ministry of Public Health and the Pan American Health Organization (PAHO) (19, 20). The national survey covered the country’s four geographic regions (Coast, Highlands, Amazon, and Insular) and distributed participants across six productive sectors, classified according to standardized epidemiological criteria to ensure risk comparability across different occupational environments (21).

The primary study employed a stratified probability sampling with proportional allocation based on the national workforce, supported by official data from the National Institute of Statistics and Censuses (INEC) and sectoral ministries (22–24). Recruitment was executed at primary healthcare centers and community spaces, integrating snowball sampling as a complementary strategy to ensure representativeness in hard-to-reach areas.

Starting from an original response rate of 97.4%, we applied strict inclusion criteria for this secondary analysis: actively employed adults (age ≥18 years) with complete survey records. The final analytical sample comprised 1,807 workers distributed across: agriculture (*n* = 534), the informal sector (*n* = 451), mining (*n* = 411), and construction (*n* = 411).

We deliberately excluded the healthcare and fishing sectors from the analytical sample for methodological reasons. The healthcare sector was omitted because the sample consisted predominantly of newly graduated rural physicians with less than 1 year of tenure, introducing a severe latency bias for chronic respiratory outcomes. The fishing sector was excluded to prevent spatial misclassification bias, as offshore work lacks spatial overlap with terrestrial cantonal satellite pixels (Supplementary Table 2).

### 2.2 Data collection and instruments

Data were collected through face-to-face interviews between September 15 and November 24, 2021, using the ArcGIS Survey123 platform (25). Fieldwork was conducted by rural physicians in the 221 cantons of Ecuador. Interviews took place at healthcare centers and active worksites without employer mediation.

Outcomes were strictly operationalized using ECRHS criteria (26): “asthma” was defined as self-reporting an asthma attack in the last 12 months; “wheezing” as any chest wheeze in the last 12 months; “chronic cough” and “chronic phlegm” as symptoms present on most days for at least 3 months a year. To avoid conceptual overlap, the composite outcome of chronic bronchitis was omitted.

The main exposure variable was productive sector, classified from self-reported job title: agriculture (farmers, day laborers, field assistants), mining (miners, operators, technicians, supervisors, drivers, security personnel), construction (masons, laborers, installers, finishers), and informal sector (unstructured economic activities). The informal sector was strictly operationalized as workers engaged in unstructured economic activities characterized by the absence of a formal labor contract and a lack of affiliation with the national social security system. This stratum predominantly comprises street vendors exposed to traffic-related pollution, informal waste recyclers in contact with particulate matter, and unregulated manual laborers facing unmitigated dust and combustion emissions without institutional oversight or personal protective equipment. Covariates were selected from the Working Conditions and Health Survey in Latin America (24). These included sex, age (18–29, 30–39, and ≥40 years), educational level (no education, primary, secondary, and higher education), smoking status (yes/no), handling of workplace chemicals (yes/no/do not know), and inhalation of chemicals at work (yes/no/do not know).

### 2.3 Environmental pollution

We determined exposure to six atmospheric pollutants using daily satellite data from the Sentinel-5P TROPOMI mission, retrieved via Google Earth Engine. We processed Level 3 Offline (OFFL) products for the absorbing aerosol index (AAI), nitrogen dioxide (NO_2_), sulfur dioxide (SO_2_), carbon monoxide (CO), ozone (O_3_), and formaldehyde (HCHO), applying pixel-level quality filters recommended by Copernicus to mitigate measurement biases derived from cloud cover and instrument noise (16).

Using FAO cartography (27), we spatially aggregated the data for Ecuador’s 221 cantons, linking these values to individual records based on workplace. Given that these instruments retrieve tropospheric column densities rather than near-surface breathing-zone concentrations, the extracted metrics (e.g., mol/m^2^) function strictly as macro-environmental proxies for cantonal atmospheric burden. Additionally, we adjusted for structural geographic confounding factors using cantonal-level elevation data from NASA’s Shuttle Radar Topography Mission (SRTM) Digital Elevation Model (28).

Finally, to identify the most relevant exposure threshold for these ecological proxies, we calculated various spatial/temporal percentiles (P25, P50, P75, and P95) over the 12-month study period, and selected the optimal predictive threshold for each respiratory outcome using the Akaike Information Criterion (AIC) (29).

### 2.4 Statistical analysis

**Figure 1:**
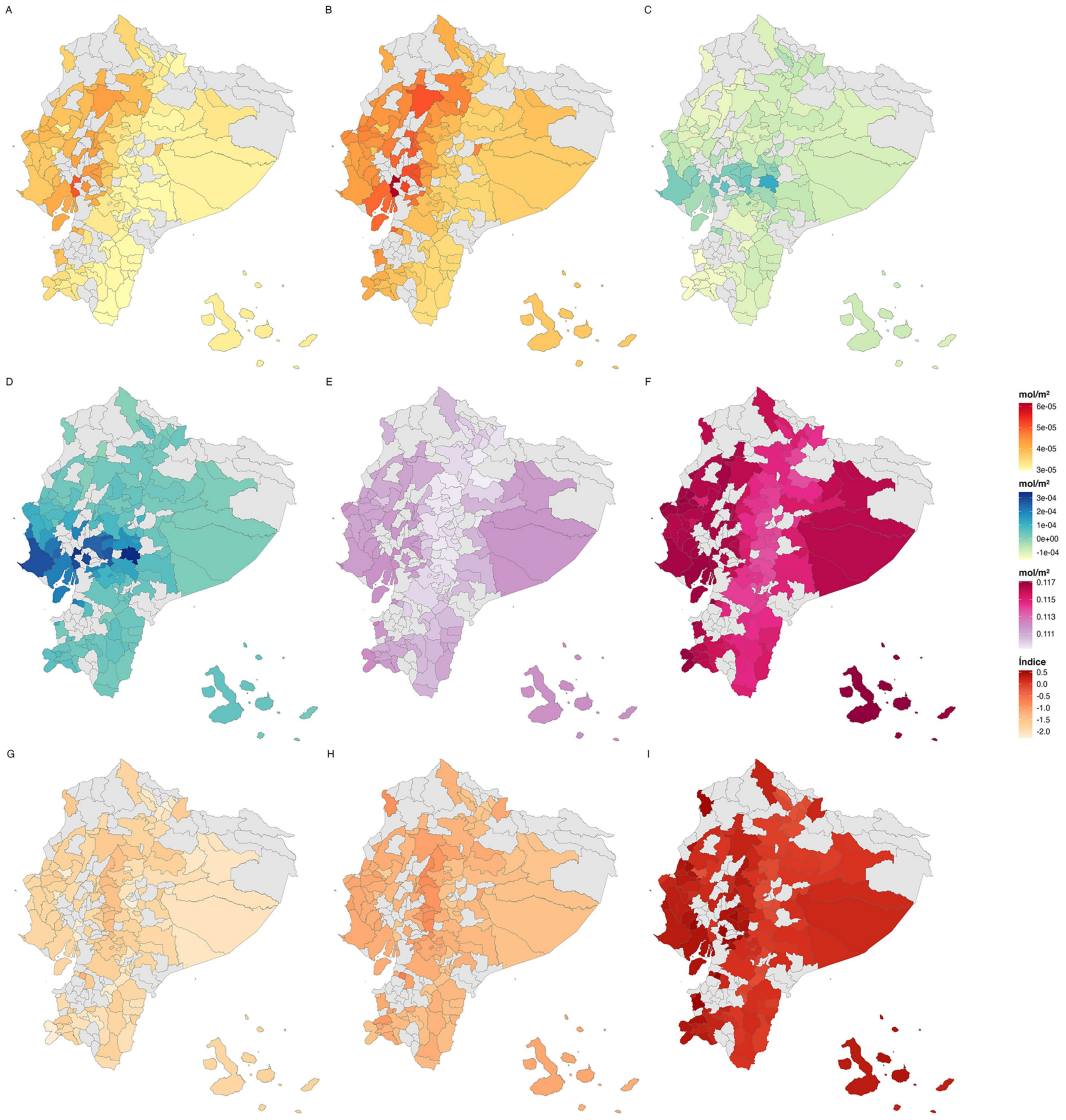
Choropleth maps illustrating significant percentiles of environmental exposure identified through logistic regression. **(A,B)** Nitrogen dioxide (NO_2_, 25th and 50th percentiles). **(C,D)** Sulfur dioxide (SO_2_, 25th and 50th percentiles). **(E,F)** Ozone (O_3_, 25th and 75th percentiles). All gaseous concentrations are expressed in mol/m^2^. **(G–I)** Absorbing Aerosol Index (AAI, 25th, 50th, and 95th percentiles) expressed as a dimensionless index. Environmental data were retrieved from the Sentinel-5P TROPOMI instrument. Cartographic note: The polygons corresponding to the Galapagos province were spatially repositioned to the east (affine transformation) exclusively for visualization layout optimization, without altering their internal geometry or representation scale.

Environmental data were retrieved from Google Earth Engine (30) and processed in R (version 2026.05.1). The temporal window for satellite data extraction was strictly aligned with the 12-month retrospective recall period of the ECRHS symptoms (November 1, 2020, to November 30, 2021). For each of the 221 Ecuadorian cantons, we calculated the spatial average altitude and multiple spatial/temporal concentration percentiles (p25, p50, p75, and p95) for O₃, NO_2_, SO_2_, and the Absorbing Aerosol Index (AAI) (Figure 1). To prevent multicollinearity, we conducted an initial screening using Spearman rank correlation; consequently, carbon monoxide (CO) and formaldehyde (HCHO) were excluded due to high variance inflation potential, while the aforementioned pollutants were retained for subsequent modeling.

We conducted a bivariate analysis to determine symptom prevalence across sociodemographic, occupational, and environmental variables. Statistical differences were evaluated using the Chi-square or Fisher’s exact test for qualitative variables, and the Mann–Whitney *U* test for continuous variables. To rigorously control for multiple hypothesis testing and minimize Type I error inflation, raw *p*-values were adjusted using the Benjamini-Hochberg procedure (31). Statistical significance for all descriptive analyses was strictly defined as an FDR-adjusted *p*-value <0.05.

To estimate adjusted Odds Ratios (aORs) and 95% confidence intervals (CIs), we performed multivariable analyses. Continuous environmental predictors were standardized using *z*-scores to facilitate interpretation (8). To determine the most representative exposure-response fit for each respiratory outcome, the optimal percentile (p25–p95) for each pollutant was empirically selected via the Akaike Information Criterion (AIC) (32). We utilized Firth’s penalized maximum likelihood regression to mitigate sparse-data bias and resolve quasi-complete separation observed in specific occupational strata (33). This regularization method ensured finite, stable parametric estimates while retaining the entire analytical sample within the models, which were simultaneously adjusted for sociodemographic, occupational, and geographic covariates.

### 2.5 Ethics approval and consent to participate

This study used a fully anonymized secondary database. As there was no direct human interaction, informed consent was not applicable. The study protocol received formal exemption from the Bioethics Committee of Universidad de Las Americas (UDLA; code 2024-EXC-006) (34), in accordance with applicable national and international regulations for research using anonymized secondary data.

## 3 Results

### 3.1 Bivariate analysis

The bivariate analysis revealed significant associations between sociodemographic, occupational, and geographic characteristics and respiratory symptoms (Table 1). Females presented a higher prevalence of chronic cough compared to males (16% vs. 12%, *p* = 0.04), alongside a higher frequency of asthma (11% vs. 7%, *p* = 0.01). Age ≥40 years was associated with increased chronic phlegm (17%, *p* = 0.01). In the occupational domain, prolonged employment duration (61–600 months) was linked to an increase in all respiratory symptoms (*p* < 0.001). The informal sector recorded the highest prevalences of asthma (21%), wheezing (6%), chronic cough (24%), and chronic phlegm (21%) compared to the construction sector, which served as the reference category (*p* < 0.001). Notably, a complete lack of knowledge regarding the risks of chemical handling and direct inhalation of dusts/aerosols yielded the highest symptom prevalences across the study population (*p* < 0.001). Additionally, the analysis demonstrated a statistically significant inverse relationship between canton-level altitude and the presence of chronic phlegm (*p* = 0.04).

**Table 1:** Prevalence of respiratory symptoms by occupational exposure and satellite environmental data.

Variable	Asthma	*p*	Wheezing	*p*	Chronic cough	*p*	Chronic phlegm	*p*
Sex, *n*%		0.013*		0.291		0.037*		0.385
Male	83 (6.6)		28 (2.2)		147 (11.6)		183 (14.5)	
Female	58 (10.7)		18 (3.3)		85 (15.7)		69 (12.7)	
Age, *n*%		0.805		0.535		0.314		0.012*
18–29 years	41 (7.5)		10 (1.8)		69 (12.6)		58 (10.6)	
30–39 years	40 (7.3)		15 (2.7)		61 (11.1)		75 (13.6)	
40 years or older	60 (8.5)		21 (3.0)		102 (14.4)		119 (16.8)	
Work experience, *n*%		<0.001*		<0.001*		<0.001*		<0.001*
0–12 months	35 (3.5)		12 (1.2)		83 (8.4)		84 (8.5)	
13–60 months	47 (7.7)		17 (2.8)		76 (12.5)		97 (15.9)	
61–600 months	59 (28.1)		17 (8.1)		73 (34.8)		71 (33.8)	
Sector, *n*%		<0.001*		<0.001*		<0.001*		<0.001*
Agriculture	23 (4.3)		7 (1.3)		64 (12.0)		70 (13.1)	
Construction	11 (2.7)		5 (1.2)		27 (6.6)		31 (7.5)	
Mining	13 (3.2)		5 (1.2)		32 (7.8)		56 (13.6)	
Informal sector	94 (20.8)		29 (6.4)		109 (24.2)		95 (21.1)	
Handles chemicals, *n*%		<0.001*		<0.001*		<0.001*		<0.001*
No	55 (4.3)		20 (1.6)		112 (8.8)		136 (10.6)	
No knowledge	82 (26.4)		26 (8.4)		82 (26.4)		75 (24.1)	
Yes	4 (1.8)		0 (0.0)		38 (17.5)		41 (18.9)	
Inhalation of chemicals, *n*%		<0.001*		<0.001*		<0.001*		<0.001*
No	34 (3.8)		10 (1.1)		80 (9.0)		103 (11.6)	
No knowledge	81 (25.4)		26 (8.2)		87 (27.3)		76 (23.8)	
Yes	26 (4.3)		10 (1.7)		65 (10.9)		73 (12.2)	
Satellital variables, mean (SD)								
Altitude (m)	1769.2 (1362.3)	0.823	1883.1 (1340.4)	0.514	1712.5 (1316.3)	0.685	1582.0 (1323.5)	0.037*
NO_2_ (mol/m^2^) p25	0.15 (1.07)	0.045*	0.17 (1.16)	0.294	0.23 (1.06)	<0.001*	0.28 (1.09)	<0.001*
NO_2_ (mol/m^2^) p50	0.15 (1.06)	0.048*	0.20 (1.19)	0.291	0.23 (1.06)	<0.001*	0.28 (1.09)	<0.001*
NO_2_ (mol/m^2^) p75	0.13 (1.06)	0.071	0.21 (1.21)	0.291	0.22 (1.06)	<0.001*	0.27 (1.09)	<0.001*
NO_2_ (mol/m^2^) p95	0.11 (1.06)	0.117	0.19 (1.20)	0.291	0.19 (1.07)	0.006*	0.25 (1.11)	<0.001*
SO_2_ (mol/m^2^) p25	0.32 (1.17)	<0.001*	0.21 (1.21)	0.276	0.23 (1.14)	0.001*	0.25 (1.12)	<0.001*
SO_2_ (mol/m^2^) p50	0.30 (1.22)	0.108	0.14 (1.19)	0.913	0.28 (1.15)	<0.001*	0.30 (1.13)	<0.001*
SO_2_ (mol/m^2^) p75	0.27 (1.17)	0.108	0.11 (1.14)	0.942	0.24 (1.09)	<0.001*	0.30 (1.13)	<0.001*
SO_2_ (mol/m^2^) p95	0.07 (1.03)	0.282	0.08 (1.44)	0.535	0.05 (0.92)	0.010*	0.15 (1.12)	<0.001*
Ozone (mol/m^2^) p25	−0.00 (1.15)	0.723	−0.14 (1.04)	0.712	−0.03 (1.12)	0.992	0.10 (1.10)	0.035*
Ozone (mol/m^2^) p50	0.06 (1.15)	0.923	−0.02 (1.10)	0.794	0.05 (1.14)	0.997	0.16 (1.12)	0.052
Ozone (mol/m^2^) p75	0.06 (1.15)	0.970	−0.00 (1.14)	0.712	0.09 (1.14)	0.840	0.18 (1.14)	0.026*
Ozone (mol/m^2^) p95	0.05 (1.16)	0.923	−0.04 (1.11)	0.622	0.06 (1.14)	0.992	0.17 (1.16)	0.035*
AAI p25	−0.13 (0.89)	0.071	−0.15 (0.91)	0.291	0.04 (0.88)	0.406	0.04 (0.95)	0.700
AAI p50	−0.14 (0.94)	0.080	−0.19 (0.98)	0.276	0.05 (0.90)	0.210	0.06 (0.98)	0.166
AAI p75	−0.14 (1.00)	0.056	−0.22 (1.01)	0.194	0.04 (0.94)	0.379	0.06 (1.01)	0.166
AAI p95	−0.15 (1.09)	0.082	−0.23 (1.10)	0.194	−0.03 (0.99)	0.822	0.08 (1.07)	0.097

Categorical variables are expressed as *n* (%) and compared via Chi-square or (^†^) Fisher’s exact test. Continuous variables (satellite pollutants *Z*-scores) are presented as mean (SD) and analyzed using the Mann–Whitney *U* test. *Indicates statistical significance (*p* < 0.05) after Benjamini-Hochberg False Discovery Rate (FDR) correction.

Regarding environmental exposures, asthma was significantly associated with low and intermediate ambient concentrations of nitrogen dioxide (NO_2_, p25 and p50; *p* < 0.05) and sulfur dioxide (SO_2_, p25; *p* < 0.001). Conversely, asthma evidenced no statistically significant relationship with the Absorbing Aerosol Index (AAI) or ozone (O_3_) across any percentiles. Chronic cough and phlegm exhibited a strong statistical association (*p* < 0.05) with both NO_2_ and SO_2_ across all evaluated percentiles (p25–p95). Similarly, O_3_ demonstrated a significant association with chronic phlegm, specifically at its 25th, 75th, and 95th percentiles (*p* < 0.05).

**Figure 2:**
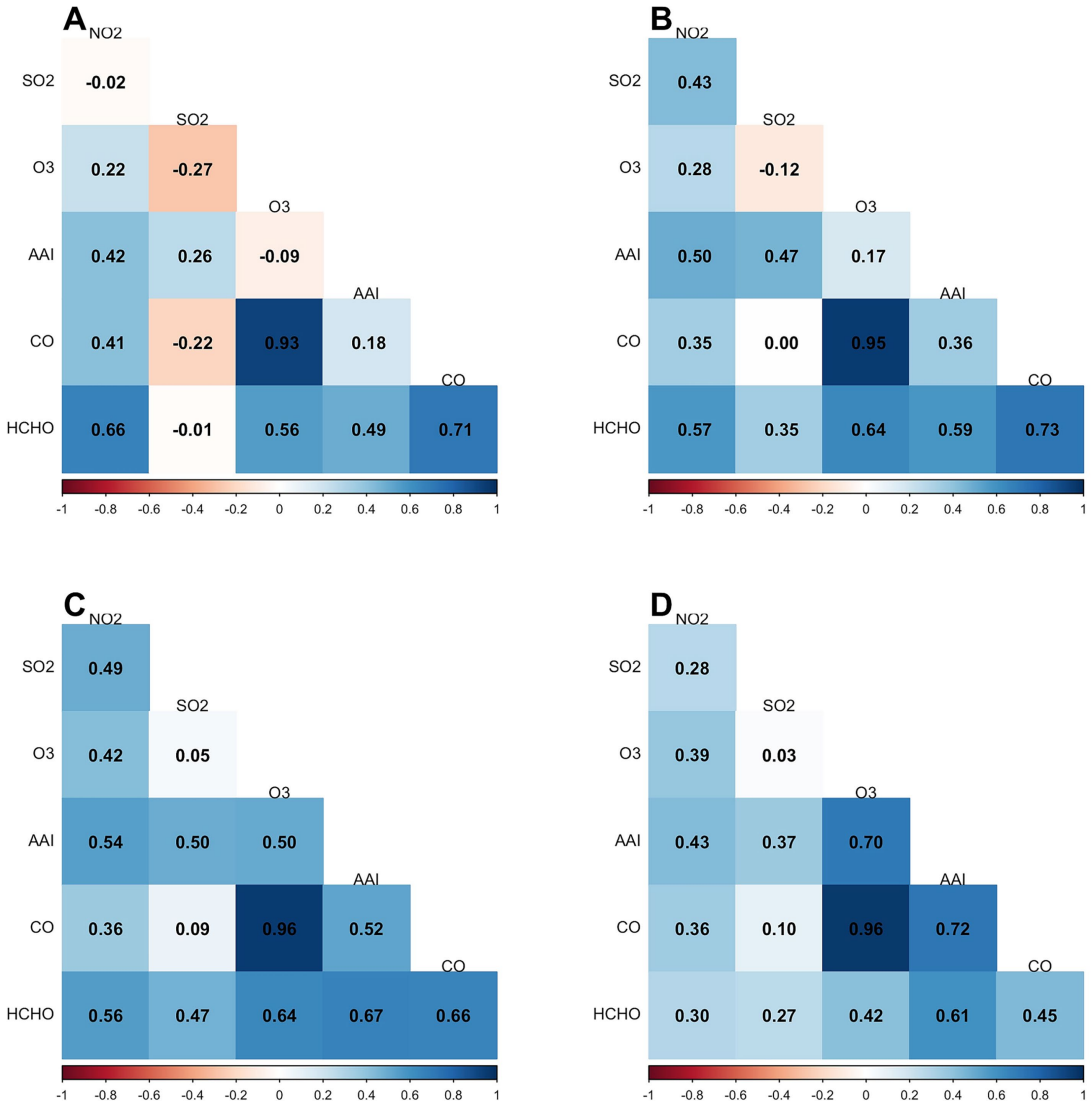
Multipollutant Spearman rank correlation matrices stratified by exposure intensity percentiles. Heatmaps display collinearity among six satellite environmental proxies at: **(A)** baseline (p25), **(B)** median (p50), **(C)** moderate peak (p75), and **(D)** extreme episodic (p95) levels. Data were transformed to μmol/m^2^ to stabilize variance. Values represent Spearman’s *ρ*.

Prior to multivariable modeling, a Spearman correlation analysis (Figure 2) detected severe multicollinearity among specific environmental indicators. Specifically, CO correlated strongly with O_3_ (*r* = 0.93–0.96 across all intensities), while HCHO exhibited high collinearity with NO_2_ and CO. To prevent variance inflation in subsequent models, we excluded CO and HCHO. The stability and structural independence of the retained predictors (NO_2_, SO_2_, O_3_, and AAI) were further confirmed via Variance Inflation Factor (VIF) analysis, ensuring all values remained below the threshold of 5 (Supplementary Table 1), thereby precluding multicollinearity bias.

### 3.2 Multivariate models

The adjusted Firth’s penalized logistic regression models (Table 2) showed a clear dose–response association regarding employment duration. Compared to newly hired workers (0–12 months), those with 13–60 months of tenure exhibited an independently higher risk of asthma (aOR = 1.99; 95% CI: 1.20–3.32) and chronic phlegm (aOR = 1.66; 95% CI: 1.19–2.32). In the stratum with over 60 months of employment, associations were observed for all evaluated outcomes: asthma (aOR = 3.51; 95% CI: 1.98–6.28), wheezing (aOR = 3.93; 95% CI: 1.60–9.92), chronic cough (aOR = 2.60; 95% CI: 1.68–3.98), and chronic phlegm (aOR = 2.70; 95% CI: 1.76–4.15).

**Table 2:** Adjusted Firth penalized logistic regression models identifying occupational, geographic, and environmental predictors of respiratory symptoms.

Variable	Asthma	Wheezing	Chronic cough	Chronic phlegm
Satellite pollutants				
NO_2_ (mol/m^2^) p95	0.88 (0.69–1.11)	1.02 (0.71–1.45)	1.06 (0.88–1.26)	1.24 (1.04–1.48)*
SO_2_ (mol/m^2^) p50	1.06 (0.87–1.27)	—	1.18 (1.02–1.35)*	1.27 (1.11–1.45)*
SO_2_ (mol/m^2^) p95	—	0.99 (0.71–1.26)	—	—
Ozone (mol/m^2^) p25	1.21 (0.70–2.10)	0.87 (0.36–2.07)	0.74 (0.49–1.11)	1.00 (0.67–1.48)
AAI p25	0.93 (0.73–1.18)	0.83 (0.57–1.19)	1.16 (0.98–1.38)	1.00 (0.85–1.17)
Geographic factor				
Altitude (km)	0.78 (0.66–0.93)*	0.86 (0.66–1.11)	0.89 (0.78–1.00)	0.87 (0.77–0.99)
Sex				
Male	1	1	1	1
Female	0.69 (0.42–1.12)	0.61 (0.29–1.28)	0.91 (0.63–1.32)	0.67 (0.45–0.98)
Age				
18–29 years	1	1	1	1
30–39 years	0.94 (0.55–1.60)	1.69 (0.72–4.07)	0.83 (0.56–1.24)	1.22 (0.83–1.81)
40 years or older	0.90 (0.55–1.49)	1.71 (0.78–3.96)	0.92 (0.64–1.33)	1.34 (0.93–1.93)
Sector				
Construction	1	1	1	1
Agriculture	3.89 (1.50–10.56)*	1.71 (0.40–7.76)	1.75 (0.94–3.32)	1.54 (0.85–2.83)
Mining	1.07 (0.43–2.68)	0.94 (0.24–3.61)	0.82 (0.45–1.51)	1.81 (1.07–3.10)*
Informal sector	12.56 (5.09–32.87)*	6.78 (1.83–27.89)*	4.14 (2.26–7.74)*	2.53 (1.39–4.67)*
Work experience				
0–12 months	1	1	1	1
13–60 months	1.99 (1.20–3.32)*	2.18 (0.98–5.01)	1.39 (0.97–1.98)	1.66 (1.19–2.32)*
61–600 months	3.51 (1.98–6.28)*	3.93 (1.60–9.92)*	2.60 (1.68–4.00)*	2.70 (1.76–4.15)*
Handles chemicals				
No	1	1	1	1
No knowledge	2.06 (1.06–3.99)*	1.96 (0.69–5.64)	1.40 (0.82–2.35)	1.69 (1.01–2.81)
Yes	0.23 (0.07–0.61)*	0.07 (0.00–0.61)*	1.56 (0.95–2.55)	1.66 (1.03–2.64)
Inhalation of chemicals				
No	1	1	1	1
No knowledge	3.58 (1.79–7.17)*	3.86 (1.23–12.09)	2.17 (1.29–3.62)*	1.31 (0.78–2.17)
Yes	2.63 (1.37–5.06)*	3.38 (1.17–9.87)	1.66 (1.09–2.53)	1.04 (0.71–1.53)
Smoking for more than 1 year				
No	1	1	1	1
Yes	3.50 (2.15–5.75)*	1.81 (0.81–3.96)	3.04 (2.12–4.37)*	3.46 (2.49–4.83)*

aOR, adjusted odds ratio; CI, confidence interval. For satellite pollutants, the percentile (p25, p50, p75, or p95) that demonstrated the optimal predictive fit according to the Akaike Information Criterion (AIC) is reported. The aORs for these environmental variables reflect the change in risk associated with a one-standard-deviation increase (*z*-score standardization). Estimates were calculated using Firth’s penalized multivariable logistic regression models to correct for quasi-complete data separation in occupational strata. All listed variables, including canton-level altitude, were adjusted simultaneously. (*) Indicates statistical significance (FDR-adjusted *p* < 0.05).

When evaluating productive sectors (reference: construction), the informal sector showed the strongest association with symptoms, with notably increased odds for asthma (aOR = 12.56; 95% CI: 5.09–32.87), wheezing (aOR = 6.78; 95% CI: 1.83–27.89), chronic cough (aOR = 4.14; 95% CI: 2.26–7.74), and chronic phlegm (aOR = 2.53; 95% CI: 1.39–4.67). The agricultural sector was a significant risk factor exclusively for asthma (aOR = 3.89; 95% CI: 1.50–10.56).

Behavioral and chemical exposure variables showed consistent associations across models. Smoking >1 year was associated with asthma (aOR = 3.50; 95% CI: 2.15–5.75), chronic cough (aOR = 3.04; 95% CI: 2.12–4.37), and chronic phlegm (aOR = 3.46; 95% CI: 2.49–4.83). The direct inhalation of workplace chemicals was a critical determinant: conscious exposure (“Yes”) increased the risk of asthma (aOR = 2.63; 95% CI: 1.37–5.06), while a complete lack of risk awareness (“No Knowledge”) yielded higher odds for asthma (aOR = 3.58; 95% CI: 1.79–7.17), wheezing (aOR = 3.86; 95% CI: 1.23–12.09), and chronic cough (aOR = 2.17; 95% CI: 1.29–3.62).

Crucially, controlling for geographic confounders revealed that canton-level altitude acted as an independent protective factor against asthma (aOR = 0.78; 95% CI: 0.66–0.93). Regarding environmental pollutants selected via the Akaike Information Criterion (AIC), nitrogen dioxide (NO_2_) at the 95th percentile (p95) emerged as an independent risk factor for chronic phlegm (aOR = 1.24; 95% CI: 1.04–1.48). Additionally, sulfur dioxide (SO_2_) at the 50th percentile (p50) showed a significant association with both chronic cough (aOR = 1.18; 95% CI: 1.02–1.35) and chronic phlegm (aOR = 1.27; 95% CI: 1.11–1.45). In these rigorously adjusted models, neither ozone (O_3_) nor the absorbing aerosol index (AAI) exhibited statistically significant independent associations with the respiratory outcomes.

## 4 Discussion

Our study revealed a multi-symptomatic association between informal employment and respiratory symptoms, highlighting significantly elevated odds for asthma (aOR = 12.56), wheezing (aOR = 6.78), chronic cough (aOR = 4.14), and chronic phlegm (aOR = 2.53). These results are comparable to previous studies in the region; for instance, while Rozo Silva et al. (35) identified that cough was predominant and a sentinel symptom among informal waste recyclers, our findings reveal a much broader respiratory compromise. These differences suggest that respiratory symptoms are strongly associated with occupational precarity, a lack of workplace regulation, and the overlapping environmental burden in each context.

When contrasting our results regarding chemical exposure with the study by Whitworth et al. (36), we observed important similarities: both demonstrate high asthma rates among precarious workers, associated with smoking (aOR = 3.50 in our study sample) and contact with toxic substances. Notably, our study identified that the inhalation of chemicals with a total lack of knowledge regarding their risks was associated with a higher odds of asthma (aOR = 3.58) and wheezing (aOR = 3.86). This association is comparable to the notably elevated risks that Whitworth et al. reported in female workers exposed to specific irritants such as ammonia (OR = 7.5) and solvents (OR = 4.5). This underscores how the absence of training and personal protective equipment (PPE) within the informal economy may be associated with increased occupational toxicity.

Our findings suggest a concurrent burden where working and environmental conditions are cross-sectionally associated with worker vulnerability. For instance, Dushyant et al. (37) reported a significant risk of respiratory symptoms in a cohort of informal workers exposed to cement dust (OR = 7.38) and in outdoor informal workers without cement exposure (OR = 5.34), compared to indoor tailors in India. In contrast, our model indicates that in the informal sector, the respiratory symptoms burden reaches similar or greater magnitudes (aOR = 12.56 for asthma) when individual determinants—such as lack of risk awareness and smoking—converge with atmospheric pollution proxies at the cantonal level. Regarding the satellite-derived environmental burden, after adjusting for geographic confounders such as canton-level altitude, nitrogen dioxide (NO_2_) and sulfur dioxide (SO_2_) emerged as the primary independent environmental predictors, while ozone lost statistical significance. Specifically, NO_2_ (p95) was associated with chronic phlegm (aOR = 1.24), and SO_2_ (p50) was associated with both chronic cough (aOR = 1.18) and phlegm (aOR = 1.27). Wafula et al. (38) and Kawano et al. (39) documented strong associations of acute respiratory infections in Uganda and phlegm in Senegal with high levels of particulate matter (PM_2.5_) and nitrogen dioxide (NO_2_), respectively. However, after adjusting for canton-level altitude in our multivariable models, tropospheric ozone (O_3_) lost its statistical significance. Nitrogen dioxide (NO_2_) and sulfur dioxide (SO_2_) were instead identified as the strongest macro-environmental predictors associated with respiratory impairment at the cantonal level. Rather than ultraviolet catalysis of ozone, this robust association suggests that the unregulated combustion of fossil fuels and dense urban traffic generate a constant primary pollutant burden (NO_2_ and SO_2_). When evaluated through a One Health perspective, these satellite-derived environmental proxies highlight how regional atmospheric degradation converges with occupational vulnerabilities, particularly affecting informal outdoor workers who lack respiratory protection.

From a multi-systemic perspective, the innovative use of high-resolution satellite data (Sentinel-5P) allowed us to identify how the macro-environmental burden interacts with occupational chemical toxicity at the cantonal level. Specifically, satellite telemetry identified cross-sectional associations suggesting that exposure to ambient pollutants such as nitrogen dioxide (NO₂) and sulfur dioxide (SO_2_), coupled with the unprotected direct inhalation of chemicals, promotes a state of severe oxidative stress, chronic inflammation, and airway remodeling—mechanisms well-documented as triggers for respiratory exacerbations and obstructive symptoms (16, 38, 39). The ability to cross-reference these readings with a highly heterogeneous population sample, while rigorously adjusting for geographic confounders like altitude, explains the phenotypic differences compared to previous research focused on isolated occupational niches (35, 37). Beyond these biological mechanisms, our findings evidence a severe structural failure: precarious informal work operates within a regulatory vacuum devoid of interventions. This marginalization is intimately linked to a complete lack of knowledge regarding the risks associated with chemical inhalation and results in an absolute lack of engineering controls and respiratory protection. Therefore, as a consequence of the strong associations identified, there is an urgent need to address this crisis through a combined management approach integrating industrial hygiene, public health, and environmental health. Within this integrative framework, future longitudinal studies are required to inform the design of public policies, merging continuous aerospace monitoring with direct occupational prevention strategies for the most vulnerable populations (36, 37).

A primary strength of this study lies in the use of a national database collected by the Ecuadorian Ministry of Public Health, which enabled us to execute large-scale comparisons across diverse labor contexts. This design provides significant geographical representativeness, highlighting the respiratory health inequalities observed among different productive sectors. Furthermore, we simultaneously integrated sociodemographic, occupational, geographic (canton-level altitude), and satellite-derived environmental telemetry data (Sentinel-5P). Although the spatial resolution of the satellite data captures cantonal macro-environmental proxies rather than individualized dosimetry, this approach allows for the evaluation of the broader ecological context of the workforce. By articulating regional environmental determinants with individual-level factors, our study adopts a robust One Health perspective, providing insights into how regional atmospheric dynamics are associated with occupational respiratory health inequities.

Additionally, respiratory symptoms were collected using the European Community Respiratory Health Survey (ECRHS), an internationally validated clinical instrument that ensures high epidemiological data quality and comparability. Likewise, the application of robust and advanced statistical methods—including Firth’s penalized logistic regression to handle quasi-complete data separation, the Akaike Information Criterion (AIC) for the optimal selection of environmental exposures, and false discovery rate (FDR) adjustments—substantially strengthens the internal validity of the identified associations.

Despite its strengths, this study presents certain limitations that must be acknowledged. First, the cross-sectional design restricts the ability to establish directional causal relationships between exposures and the onset of respiratory symptoms. Crucially, the assignment of environmental exposures at the cantonal level introduces potential ecological misclassification. The Sentinel-5P data serves as ecological proxies for ambient air quality rather than direct measurements of individual-level personal dosimetry. Therefore, our findings reflect macro-environmental risks—suggesting that vulnerable workers operate in regions with higher pollutant densities—rather than confirming precise individual exposures. We acknowledge that spatial resolution at the canton level does not accurately capture individual micro-exposure. Although we implemented concentration percentiles to obtain a more robust estimate of environmental exposure levels, these macro-environmental metrics do not constitute absolute individual dose thresholds. Actual exposure varies significantly depending on specific work schedules, lack of protective equipment, and individual occupational microenvironments.

### 4.1 Limitation due to spatial aggregation

A critical limitation of this study is the potential for ecological fallacy arising from spatial aggregation. Environmental exposure data from Sentinel-5P TROPOMI, which has a native resolution of approximately 5.5 × 3.5 km, were aggregated to the cantonal level (*n* = 221). Assigning a cantonal average to each worker is not equivalent to measuring individual-level exposure (40–42). Therefore, the associations identified—such as the odds ratios for sulfur dioxide (SO_2_; aOR = 1.27 for chronic phlegm at p50)—reflect regional macro-environmental risks rather than precise individual-level causal effects. Consequently, our findings indicate that workers in cantons with higher pollutant densities have a greater statistical probability of reporting respiratory symptoms. These results should be interpreted as hypothesis-generating evidence regarding the impact of regional atmospheric burdens and not as absolute individual dose thresholds.

Furthermore, the models may be subject to residual confounding. Key unmeasured variables, including domestic biomass fuel exposure, passive smoking, a history of prior respiratory diseases (such as childhood asthma or pre-existing COPD), urban/rural residence, and household crowding, were not available in the primary dataset but likely play a substantial role in respiratory symptoms. Although we successfully controlled for macro-geographic heterogeneity by incorporating canton-level altitude, the assessment of respiratory outcomes was based on self-reported questionnaires; the absence of biological monitoring of lung function (such as spirometry) or on-site clinical diagnosis limits the objective confirmation of structural respiratory pathology. Finally, the dataset did not provide spatial tracking to capture daily mobility between discordant residential and occupational cantons, nor did it include individual sampling weights (expansion factors) to adjust for potential network bias introduced by snowball sampling in hard-to-reach areas, which limits the strict generalizability of the findings.

In conclusion, this study suggests that workers in the informal sector exhibit significantly higher odds of experiencing respiratory symptoms, particularly asthma and wheezing, compared to formal sectors. These respiratory symptoms are strongly associated with the interaction of severe occupational vulnerabilities—such as the direct inhalation of chemicals without preventive knowledge—and behavioral factors, with smoking emerging as a consistent factor associated with asthma, chronic cough, and chronic phlegm. From a geospatial perspective, after adjusting for the protective effect of altitude, nitrogen dioxide (NO_2_) and sulfur dioxide (SO_2_) showed the strongest associations at the cantonal level among the evaluated pollutants, highlighting a significant link between regional atmospheric burden and chronic respiratory symptoms in the study population. Future research should first focus on validating satellite-derived proxies against ground monitors in Ecuador before considering their integration into routine epidemiological surveillance systems. Furthermore, as a consequence of the strong associations identified in this study, there is an urgent need to strengthen respiratory protection strategies within the informal sector through practical interventions, such as municipal subsidies for personal protective equipment (PPE) and community-based occupational training programs, implement targeted smoking prevention programs, and promote inter-sectoral policies that merge industrial hygiene with environmental health, to reduce labor inequalities and mitigate the combined risks faced by the Ecuadorian workforce.

## Data Availability

The raw data supporting the conclusions of this article will be made available by the authors, without undue reservation.
